# Training Community Members in Public Health Research: Development and Implementation of a Community Participatory Research Pilot Project

**DOI:** 10.1089/heq.2018.0043

**Published:** 2018-10-12

**Authors:** Goldie Komaie, Melody Goodman, Angela McCall, Gloria McGill, Chavelle Patterson, Cassandra Hayes, Vetta Sanders Thompson

**Affiliations:** ^1^ Division of Public Health Sciences, Department of Surgery, Washington University School of Medicine (WUSM), St. Louis, Missouri.; ^2^ College of Global Public Health, New York University, New York, New York.; ^3^ Community Research Fellows Training Program, Program for the Elimination of Cancer Disparities at Siteman Cancer Center, Division of Public Health Sciences, WUSM, St. Louis, Missouri.; ^4^ Brown School, Washington University in St. Louis, St. Louis, Missouri.

**Keywords:** community-based participatory research, community engagement, research literacy

## Abstract

**Purpose:** Community-based training in public health research can build capacity for community-based participatory research (CBPR) and foster health partnerships between academics and stakeholders. We describe a community-academic partnership developed from a 15-week program, the Community Research Fellows Training (CRFT), designed to increase research literacy and facilitate equitable relationships in community/researcher collaborations and partnerships. The article provides a description of a community and faculty collaboration to conduct a participatory pilot research project that followed program completion.

**Methods:** Four CRFT program alumni formed a community research team and selected a faculty mentor. After a request for proposal release, the team developed a pilot research proposal that addressed a concern for mental health among women experiencing economic stress. After completion of the pilot research, the community researchers elected to participate in two dissemination efforts, including a manuscript reflecting on their research experience. Team successes, challenges, and recommendations for future training are discussed.

**Results:** Each member of the CRFT pilot research team reflects on how training prepared community members to conduct CBPR research through development and implementation of a pilot research project. Community researchers gained experience in grant proposal development, choosing appropriate health interventions, conducting in-person surveys and telephone interviews, and disseminating study findings.

**Conclusions:** Providing training in public health research before community/researcher collaboration can increase community capacity to engage in research as equitable partners in research question development, study design, and data interpretation and dissemination. The project success suggests that this and similar programs maximize the potential of community-academic health partnerships to address health disparities.

## Introduction

Community-based participatory research (CBPR) is believed to promote community participation in research, which may increase the accuracy of the data collected and how those data are interpreted.^1^ It is believed that CBPR strategies allow findings and resulting interventions from the collaborative work to have increased acceptance, adoption, and sustainability within the community, because of the community's awareness that their input and perspectives influenced the efforts.^1–3^ In addition, it is believed that CBPR empowers and changes people's perceptions of themselves and what they can accomplish, stimulating capacity for change.^4^

Improving research literacy, or the basic knowledge of research methods, study design, and research terminology, is needed to increase community research capacity.^5^ Successful academic-community partnerships cannot be fully realized if community members lack sufficient research literacy to feel comfortable offering their perspectives or challenging researcher assumptions about appropriate questions and methods for their community. Research institutions can implement research literacy training as a component of capacity building to position community partners for full participation in the research process.^5–8^ Community partners are then able to promote co-learning—or sharing and transferring knowledge, skills, capacity, and power so that findings and knowledge benefit all partners—by inviting researchers to participate with them as they describe the issues that affect their communities and their ideas to address the issues of greatest concern.^3,9^ When opportunities for pilot research are made available, the institutional implementation of research literacy training and community partner/researcher collaboration on community-initiated research provides both parties with the knowledge and opportunity to fully implement CBPR.^6,8^

In an effort to foster community-academic relationships and increase research literacy among community stakeholders in St. Louis, the Division of Public Health Sciences at Washington University School of Medicine, and the Siteman Cancer Center, began the Community Research Fellows Training (CRFT) program. The CRFT is a comprehensive 15-week evidence-based public health training that promotes engaging underserved populations in the research enterprise. The CRFT program has been shown to provide a conducive learning environment for increasing participants' knowledge of public health research between baseline and follow-up.^5–8^ The overall goal of the program is to equip community members with the tools and resources to examine and address health disparities that exist among communities of color and medically underserved populations in the region.

To extend learning from the classroom to practice and encourage community-academic partnership, possible pilot research opportunities have been made available to CRFT Fellows. The pilot projects were intended to benefit Fellows and their communities or community-based organizations (CBOs). Although a description of demographic characteristics of cohort I pilot research teams has been described in greater detail elsewhere,^6^ this article is an effort to describe the CBPR process of developing and completing a community-led pilot research project. Specifically, we describe the process of collaboration among cohort III alumni to conduct a pilot research project to address mental health among African Americans in St, Louis, including development of a research proposal, a timeline of pilot research activities, and the community researcher's reflections about the research experience.

## Methods

The President and CEO of the GrassROOTS Community Foundation (GCF) pledged to support CRFT III pilot research. The CRFT Program Director, in consultation with the GCF President, developed a request for proposal (RFP) that aligned with the GCF mission. The RFP stated that projects should address and improve health outcomes among African American women and girls.^10^ Proposals were to be evaluated based on the project's evidence of a strategic plan that engaged community members, promotion of academic-community collaboration, culturally appropriate approaches to meet the needs of diverse communities, and the project's impact on the specified population. An information session was held by CRFT staff where attendees received a copy of the RFP, were able to ask questions about funding and the application process, and received feedback on potential project ideas. Fellows were encouraged to work collaboratively in teams up to four members, with one member taking a leadership role as “Community Principal Investigator” (PI). The identification of a faculty mentor was the decision of the community research team. The faculty mentor's role was that of a consultant, role model, and guide, who provided advice on the formulation of the research question, inclusion and exclusion criteria for potential participants, assessment measures, and intervention strategies. In this instance and a previous effort, community members sought to work with faculty members who led CRFT sessions.^6^ Representatives from two groups, one of which was funded and a second group that did not submit a proposal, attended.

The community research team comprised four African American women aged 25–65 years old. Two of the community researchers worked full-time, one was retired, and one worked part-time. None of the team had prior experience conducting research, and they had never participated in CBPR initiatives. They selected a CRFT faculty mentor, also an African American woman, whose research expertise in mental health aligned with the proposed project.

Before the proposal was submitted to GCF, the project team submitted a draft of the grant proposal to the Patient Research Advisory Board (PRAB) for evaluation and feedback. The PRAB comprised CRFT alumni and is a community research review board that advocates for community health concerns and projects with community benefit.^5^ The Community PI made an oral presentation and received oral feedback from the group. In addition, three members of the PRAB and two CRFT faculty provided written feedback on the proposal. The team was encouraged to narrow the study population, develop inclusion and exclusion criteria for study participants, and develop a follow-up component to the study.

After initial review of the proposal submitted, GCF recommended that the study team revise and resubmit the proposal. The proposal was funded after the second GCF review. The main goal of the pilot research project was to investigate levels of stress experienced by unemployed African American mothers and whether educational materials about the effects of stress related to unemployment improved their recognition of stress, stress management, and knowledge of when to seek mental health services. Table 1 illustrates the timeline of key project milestones.

**Table 1.** **Timeline of Community Research Fellows Training Pilot Project Milestones**

Milestones	Date
CRFT cohort III graduation	August 2015
RFP for pilot projects released	September 2015
Submission of pilot grant proposals	October 2015
Grant proposal revised and resubmitted	January 2016
Pilot project awarded funding	February 2016
IRB approval from Washington University	June 2016
Data collection	June–September 2016
Data analysis	October 2016
Abstract submission	October 2016
Poster presented	November 2016

CRFT, Community Research Fellows Training; IRB, Institutional Review Board; RFP, request for proposal.

The CRFT program manager drafted an Institutional Review Board (IRB) protocol application that was approved by the faculty mentor and then submitted for IRB review. Each community researcher was added as a research team member to the IRB application. To accomplish this addition, the IRB required that each community researcher submit a resume and human subjects training certification (which they had already obtained in CRFT). The Human Research Protections Office at Washington University in St. Louis approved the study.

Between June and August 2016, 50 participants (African American unemployed mothers, 21 to 54 years) were recruited to participate in the pilot research. The majority (86%) of participants had an annual income of less than $10,000, 50% were unemployed for 6 months or less, and 88% had more than a high school or higher level of education (Table 2). Participants completed the informed consent process, a presurvey, and then watched an educational video from the National Alliance on Mental Illness. Participants completed a postsurvey, received mental health and community resources, and 22 participants completed the 4-week follow-up survey to determine use of mental health services.

**Table 2.** **Pilot Study Participant Demographics (*n*=50)**

	*n*	Percent
Race		
Black/African American	50	100
Household income		
No income	19	38
Under $10,000	24	48
$10,000 to $19,999	3	6
$20,000 to $29,999	1	2
Missing	3	6
Children between 3 and 16		
1	12	24
2	21	42
3	7	14
4 or more	10	21
Unemployed length		
0–3 months	17	34
3–6 months	8	16
6–12 months	6	12
1–3 years	9	18
More than 3 years	9	18
Missing	1	2
Education level		
Less than HS	5	10
HS or equivalent	15	30
Vocational/tech school	7	14
Some college	15	30
Bachelor's degree	7	14
Missing	1	2

HS, high school.

Due to IRB requirements for data security, data were located at the University on secure servers. For this reason, CRFT staff completed quantitative data analyses. Results showed that almost all participants (98%) reported having experienced at least one of the seven stress symptoms since being unemployed. Most (88%) of the women reported having experienced at least 1 of the 13 mental health warning signs due to stress related to unemployment. The majority of participants (59%) did not report using any mental health services. Data were reviewed and discussed by the community research team. The research team and faculty mentor discussed questions that the follow-up interviews might inform and aided in the coding of the follow-up interviews, which were completed by the faculty mentor and a research assistant. A community research team member created additional tables and charts to summarize the data.

Once the pilot research was completed, the community research team and faculty mentor met to debrief on the project and discuss next steps. The faculty mentor queried interest in dissemination opportunities. The team decided to work with the faculty mentor and CRFT faculty and staff on a paper that addressed not only the pilot project but also their research experiences. The faculty mentor developed a series of questions that were used as prompts to assist the community researchers in reflecting on their experiences. The community researchers submitted their written responses to these questions, which are used to discuss the pilot project development, process, and benefits. The faculty mentor read and summarized the responses. Across the four team members, four consistent themes emerged: (1) their reasons for participating in the pilot research, (2) successes, (3) challenges, and (4) rewards of participation. The team members reviewed the paper and the integration of their perspectives into the manuscript.

## Results

The community researchers described how they formed their team and their motivation for selecting mental health as a study topic. They described their concern about the level of stigma around mental health in the African American community:

“When [the Community PI] first mentioned mental health as a research topic, I was immediately intrigued and knew that I wanted to partner with her. In the African American community, there is a stigma around mental health and wellness. Mental illness often goes undiagnosed in African American communities compared to other communities and African Americans are often left to heal and cope on their own…for me this research topic was a great way to understand current behaviors and stigmas surround mental health in St. Louis and bring more awareness of mental health resources to African American communities.”

Once the team selected the topic, they planned weekly meetings that took place either by teleconference or in-person. The meeting times were used to conceptualize a project and narrow the focus, identify areas of the proposal that each would focus on and write up for the grant proposal sections. The team described the collaborative process of grant writing and how they learned from each other:

“I really enjoyed working with the team to prepare the proposal. Each member of our team brought different experiences and skills to the table. We discussed many research methods and strategies to approach our research question. With the help of our advisors and mentors, we were able to leverage everyone's strengths and reach a consensus on the best approach for that stage of the project.”

The team described reaching consensus after discussing various methods and intervention tools. They selected a brief informational video to show women how to recognize the symptoms of anxiety and stress, so that they could learn to manage stress better and, if needed, to use available mental health services. Educational materials on coping with stress, as well as a directory of resources, were compiled to give to study participants. One community researcher described what she hoped to accomplish:

“I wanted to learn whether unemployed African American women who had experienced stress and other symptoms of mental illness were aware of local resources and other methods to help them heal and cope. I also hoped to educate mothers on the effective strategies and methods to cope with mental illness, so that they could take steps to improve their health.”

Community members took significant responsibility for the project, including securing recruitment sites. The Community PI was employed by a CBO that administers programs to assist low-income people out of poverty conditions. The Community PI approached the Director of Program Administration for approval to recruit participants and collect data at their site, which was approved. She continued to identify recruitment locations until the IRB-approved sample size was recruited.

The community researchers discussed what they learned about the research process, highlighting aspects they found surprising, challenging, and rewarding. They discussed being surprised at the amount of time and rigor that was involved:

“Implementing the research was very eye opening for me. We had to maintain rigor to recruit enough participants… Also, because health is a personal subject, I learned how important it is for the researcher to establish trust, so the subject felt comfortable enough to be honest about their experiences.”

The value of conducting in-person interviews was evident as they learned about the need to establish trust with participants. Also, one community researcher explained how “it was helpful to be able to interact with study participants and to hear how the study impacted them.” Hearing first-hand about participants' financial and emotional struggles with unemployment reinforced the value of conducting the research.

Overall, the community research team described how the CRFT program prepared them for conducting research. The community PI explained how the program's “focus on community research prepared me for working with the public when addressing public health issues. Each week's topic was beneficial and built on connected issues within the public health field.” Another member stated that “CRFT was successful in giving us the tools we needed. Like everything else, the real learning is in the doing.”

The community researchers also reflected on some of the challenges they encountered during the proposal writing and project implementation phases of the work. In the study design, one community researcher described spending too much time independently writing; “If I had to do it again, I would engage our advisor and mentors earlier in the process of writing the research proposal.” Another challenge was with the length of the IRB review. The team was eager to get started on the project and felt frustrated with the IRB review process. Finally, the community researchers did not anticipate some of the challenges that can arise when conducting a research project that included a baseline and 4-week follow-up. The time from start to finish led to concerns about the ability to sustain participant interest. One community researcher remarked:

“Towards the end of the project, during the follow-up telephone interview stage, I felt that the participants were exhausted and deterred from continuing with the research study because most of the participants did not want to speak to us when we called. Also, the follow-up was extensive and involved coordinating our schedules to the appointment times we had scheduled with the participants…We could have shortened the research project from the time it had been implemented to the time we decided to do the follow-up survey because the participants seemed to have lost interest and were pre-occupied while we spoke with them…In the end, it was very challenging to make a connection with them.”

She describes logistical challenges such as coordinating team members' schedules with participant scheduled interviews and the difficulty in making a connection with participants over the telephone.

## Discussion

CBPR aims at involving academic and community partners in all aspects of the research process.^1–3^ In this article, we provide an example of how we developed and implemented a CBPR pilot project between academic researchers and community members who had completed a public health research training program. Based on the community researchers' experiences with the pilot project, they made recommendations that will inform CRFT curriculum modifications.

Community research pilot team members struggled mostly at the onset with developing a succinct proposal that could be accomplished in a realistic timeframe. The community researchers recommended including a mock research project that is presented at the beginning of CRFT. Each subsequent session would then develop and extend the project, beginning with the identification of the problem and community stakeholders, the appropriate research questions, selection of the methodology, and application of quantitative and qualitative methods. Being exposed to how a project is developed over the course of the CRFT program would have better familiarized them with how to develop their own project since the team struggled to narrow the scope of their project.

Data collection revealed the importance of direct research experience for understanding both human subjects' protections (e.g., documentation, security, and protocol adherence) and research procedures. The members of the community research team were initially more nervous about participant screening, recruitment, and data collection than anticipated. The faculty mentor was on-site to model how to approach potential participants, conduct the informed consent process, and administer the survey. Over time, community team members became more confident and independent and less in need of faculty mentor input.

Previous articles have demonstrated efficacy and satisfaction with the training and suggest pilot research as the next step to expand learning outcomes.^11^ A prior paper provided pilot project results; however, this is the first to discuss community researchers' perspectives on running a pilot project and lessons learned. Despite the strengths of the pilot research report, there are limitations. Clearly, we are reporting on a small case study and although it suggests opportunities for future efforts, the experiences reported may not be generalized. In addition, the report represents a summative evaluation and not a process evaluation. Finally, we cannot report on the full research experience because the Fellows were not able to complete or assist in the data analyses.

## Conclusion

Findings from this pilot project have been disseminated through an academic venue (poster presentation), community presentations presented by the Community PI, and a final report submitted to the funder and participating CBO. The pilot project extended the CRFT training experience to a real-world setting promoting community-engaged research by providing an opportunity and space to forge an academic-community partnership. The CBPR pilot project serves as an example of how a public health training program can enhance the infrastructure for community-driven projects and be mutually beneficial to both community members and academics interested in projects that address health disparities. Modifications will be made to the existing CRFT curriculum to address the challenges encountered.^10^

It is important to extend research literacy training into real-world practice. There are some things (e.g., time to IRB approval, IRB protocols, recruiting participants) that cannot be learned in the CRFT training. If we are dedicated to increasing research literacy, we must also provide opportunities for practice (e.g., pilot project funding, faculty and staff time to support implementation).

## Abbreviations Used

CBOs: community-based organizations
CBPR: community-based participatory research
CRFT: Community Research Fellows Training
GCF: GrassROOTS Community Foundation
IRB: Institutional Review Board
PI: Principal Investigator
PRAB: Patient Research Advisory Board
RFP: request for proposal

## References

[1] Wallerstein N, Duran B Community-based participatory research contributions to intervention research: the intersection of science and practice to improve health equity. Am J Public Health. 2010;100 (Suppl 1):S40–S46 20147663 10.2105/AJPH.2009.184036 PMC2837458

[2] Minkler M Community Organizing and Community Building for Health. New Brunswick, NJ: Rutgers University Press, 2003

[3] Israel BA, Schulz AJ, Parker EA, et al. Review of community-based research: assessing partnership approaches to improve public health. Annu Rev Public Health. 1998;19:173–202 9611617 10.1146/annurev.publhealth.19.1.173

[4] KU Work Group for Community Health and Development. Introduction to evaluation: community-based participatory research. In: The Community Tool Box: Evaluating Community Programs and Initiatives. Lawrence, KS: The University of Kansas. Available at https://ctb.ku.edu/en Accessed February 29, 2016

[5] Komaie G, Ekenga CC, Sanders Thompson VL, et al. Increasing community research capacity to address health disparities. J Empir Res Hum Res Ethics. 2017;12:55–66 28220721 10.1177/1556264616687639 PMC5321619

[6] Coats JV, Stafford JD, Sanders Thompson V, et al. Increasing research literacy: the community research fellows training program. J Empir Res Hum Res Ethics. 2015;10:3–12 25742661 10.1177/1556264614561959 PMC4605821

[7] D'Agostino McGowan L, Stafford JD, Thompson VL, et al. Quantitative evaluation of the community research fellows training program. Front Public Heal. 2015;3:179 10.3389/fpubh.2015.00179 PMC4504145 26236703

[8] Goodman MS, Dias JJ, Stafford JD Increasing research literacy in minority communities: CARES fellows training program. J Empir Res Hum Res Ethics. 2010;5:33–41 10.1525/jer.2010.5.4.33 PMC3177406 21133785

[9] Wallerstein NB, Duran B Using community-based participatory research to address health disparities. Health Promot Pract. 2006;7:312–323 16760238 10.1177/1524839906289376

[10] Goodman MS, Thompson VS Public Health Research Methods for Partnerships and Practice. London, United Kingdom: Taylor & Francis Group, 2018

[11] Goodman MS, Gonzalez M, Gil S, et al. Brentwood community health care assessment. Prog Community Health Partnersh. 2014;8:29–39 24859100 10.1353/cpr.2014.0017 PMC4394008

